# Effect of allopurinol in addition to hypothermia treatment in neonates for hypoxic-ischemic brain injury on neurocognitive outcome (*ALBINO*): study protocol of a blinded randomized placebo-controlled parallel group multicenter trial for superiority (phase III)

**DOI:** 10.1186/s12887-019-1566-8

**Published:** 2019-06-27

**Authors:** Christian A. Maiwald, Kim V. Annink, Mario Rüdiger, Manon J. N. L. Benders, Frank van Bel, Karel Allegaert, Gunnar Naulaers, Dirk Bassler, Katrin Klebermaß-Schrehof, Maximo Vento, Hercilia Guimarães, Tom Stiris, Luigi Cattarossi, Marjo Metsäranta, Sampsa Vanhatalo, Jan Mazela, Tuuli Metsvaht, Yannique Jacobs, Axel R. Franz, for the ALBINO Study Group, Axel R. Franz, Mario Rüdiger, Christian F. Poets, Manon Benders, Frank van Bel, Karel Allegaert, Gunnar Naulaers, Dirk Bassler, Katrin Klebermaß-Schrehof, Maximo Vento, Hercilia Guimarães, Tom Stiris, Luigi Cattarossi, Marjo Metsäranta, Sampsa Vanhatalo, Jan Mazela, Tuuli Metsvaht, Cees van Veldhuizen, Corinna Engel, Christian A. Maiwald, Gabriele von Oldershausen, Iris Bergmann, Monika Weiss, Caroline J. B. R. Wichera, Andreas Eichhorn, Michael Raubuch, Birgit Schuler, Cees K. W. van Veldhuizen, Bas Laméris, Yannique Jacobs, Roselinda van der Vlught-Meijer, Elke Griesmaier, Johannes Brandner, Marie Tackoen, Ruth Reibel, Chantal Lecart, Luc Cornette, Genevieve Malfilatre, Renaud Viellevoye, Tuuli Metsvaht, Mari-Liis Ilmoja, Pille Saik, Ruth Käär, Pille Andresson, Marjo Metsaranta, Axel R. Franz, Rolf Schloesser, Torsten Ott, Stefan Winkler, Thomas Hoehn, Norbert Teig, Michael Schroth, Ulrich H. Thome, Harald Ehrhardt, Luigi Cattarossi, Isabella Mauro, Eugenio Baraldi, Virgilio Carnielli, Giuseppe Paterlini, Marcello Napolitano, Paola Francesca Faldini, Gianluca Lista, Gianluca Visintin, Mario Barbarini, Laura Pagani, Emmanuele Mastretta, Giovanni Vento, Monica Fumagalli, Marco Binotti, Mirjam M. van Weissenbruch, Henrica L. M. van Straaten, Manon J. N. L. Benders, Kim V. Annink, Frank van Bel, Jeroen Dudink, Jan B. Derks, Inge P. de Boer, Clemens B. Meijssen, Timo R. de Haan, Linda G. van Rooij, Jacqueline L. van Hillegersberg, Minouche van Dongen, Jos Bruinenberg, Koen P. Dijkman, Marlies A. van Houten, Sophie R. D. van der Schoor, Tom Stiris, Bodil Salvesen, Moritz Schneider, Eirik Nestaas, Britt Nakstad, Jan Mazela, Lukas Karpinski, Ewa Gulczynska, Barbara Królak-Olejnik, Renata Bokiniec, Ana I. Vilan, Liliana Flores de Pinho, Claudia Ferraz, Almerinda Pereira, Rosalina Barroso, André Mendes da Graça, Teresa Tomé, Filomena Pinto, Maximo Vento, Juan Martínez Rodilla, Simón Lubián, Marta Campubri Camprubí, José Antonio Hurtado Suazo, Eva Valverde, José Ramón Fernández Lorenzo, José Martinez Orgado, Héctor Boix, Francisco Jimenez Parrilla, Maria Teresa Moral-Pumarega, Julia Maletzki, Claudia Knoepfli, Cornelia Hagmann, Sven Schulzke, Martin Stocker, André Birkenmaier, Thomas Riedel, Hans-Jörg Ehni, Annie Janvier, Georg Marckmann

**Affiliations:** 1 University Hospital Tuebingen, Calwerstr. 7, 72076 Tuebingen, Germany; 2 Universitair Medisch Centrum Utrecht, Utrecht, The Netherlands; 3 Universitätsklinikum C. G. Carus - Medizinische Fakultät der TU Dresden, Dresden, Germany; 4 University Hospitals Leuven, Leuven, Belgium; 5 UniversitaetsSpital Zuerich, Zuerich, Switzerland; 6 Medizinische Universitaet Wien, Wien, Austria; 7 Hospital Universitario y Politécnico La Fe, Valencia, Spain; 8 Centro Hospitalar Universitário São João Porto, Porto, Portugal; 9 Oslo Universitetssykehus HF, Oslo, Norway; 10 Azienda sanitaria universitaria integrata di Udine, Udine, Italy; 11 Helsinki University Hospital (HUS), Helsinki, Finland; 12 Poznan University of Medical Sciences - Department of Neonatology, Poznan, Poland; 13 Tartu University Hospital, Tartu, Estonia; 14 ACE Pharmaceuticals BV, Zeewolde, The Netherlands; 15 Center for Pediatric Clinical Studies (CPCS), University Hospital Tuebingen, Tuebingen, Germany

**Keywords:** Allopurinol, Neonatal oxygen deficiency, Hypothermia therapy, Childbirth outcome, Hypoxic-ischemic encephalopathy, Perinatal asphyxia, Brain injury, Cerebral palsy

## Abstract

**Background:**

Perinatal asphyxia and resulting hypoxic-ischemic encephalopathy is a major cause of death and long-term disability in term born neonates. Up to 20,000 infants each year are affected by HIE in Europe and even more in regions with lower level of perinatal care. The only established therapy to improve outcome in these infants is therapeutic hypothermia. Allopurinol is a xanthine oxidase inhibitor that reduces the production of oxygen radicals as superoxide, which contributes to secondary energy failure and apoptosis in neurons and glial cells after reperfusion of hypoxic brain tissue and may further improve outcome if administered in addition to therapeutic hypothermia.

**Methods:**

This study on the effects of *AL*lopurinol in addition to hypothermia treatment for hypoxic-ischemic *B*rain *I*njury on *N*eurocognitive *O*utcome (ALBINO), is a European double-blinded randomized placebo-controlled parallel group multicenter trial (Phase III) to evaluate the effect of postnatal allopurinol administered in addition to standard of care (including therapeutic hypothermia if indicated) on the incidence of death and severe neurodevelopmental impairment at 24 months of age in newborns with perinatal hypoxic-ischemic insult and signs of potentially evolving encephalopathy. Allopurinol or placebo will be given in addition to therapeutic hypothermia (where indicated) to infants with a gestational age ≥ 36 weeks and a birth weight ≥ 2500 g, with severe perinatal asphyxia and potentially evolving encephalopathy. The primary endpoint of this study will be death or severe neurodevelopmental impairment versus survival without severe neurodevelopmental impairment at the age of two years. Effects on brain injury by magnetic resonance imaging and cerebral ultrasound, electric brain activity, concentrations of peroxidation products and S100B, will also be studied along with effects on heart function and pharmacokinetics of allopurinol after iv-infusion.

**Discussion:**

This trial will provide data to assess the efficacy and safety of early postnatal allopurinol in term infants with evolving hypoxic-ischemic encephalopathy. If proven efficacious and safe, allopurinol could become part of a neuroprotective pharmacological treatment strategy in addition to therapeutic hypothermia in children with perinatal asphyxia.

**Trial registration:**

NCT03162653, www.ClinicalTrials.gov, May 22, 2017.

## Background

Neonatal hypoxic-ischemic encephalopathy (HIE) as a result of perinatal asphyxia is a major cause of death and long-term disability in term neonates. About 1–4 per 1000 live births and consequently about 5–20,000 infants per year are affected in Europe [1]. In regions with lower level perinatal care it is even more common. HIE affects about 1 million infants worldwide each year.

Up to now, the only established therapy to improve outcome in infants with HIE is therapeutic hypothermia [2, 3]. However, despite therapeutic hypothermia and modern supportive neonatal intensive care, 45–50% of the infants with moderate or severe HIE (i.e., 2500–10,000 infants per year in Europe) still die or suffer from long-term neurodevelopmental impairment (NDI) such as cerebral palsy (CP), cognitive or behavioral problems [2, 4]. Therefore, additional therapies, including pharmacotherapy, are investigated to further improve the neurodevelopmental outcome of infants with HIE.

One of the potential beneficial pharmacological interventions is allopurinol. Allopurinol is a xanthine oxidase inhibitor, which reduces the production of oxygen radicals, most importantly of superoxide [5]. Superoxide radicals damage mitochondria resulting in secondary energy failure and apoptosis affecting neurons and glial cells after reperfusion of hypoxic brain tissue, this is called reperfusion injury [6, 7]. This reperfusion injury leads to additional brain injury occurring in the hours after birth and may affect much larger areas of brain tissue than the area primarily affected during the sentinel event [7]. Superoxide production, which is reduced by allopurinol, reaches its peak within 30 min after birth and therefore early administration is important to reduce reperfusion injury [8]. Furthermore, allopurinol, especially in higher concentrations, possibly chelates non-protein bound iron and scavenges the hydroxyl free radicals [9, 10]. Allopurinol also prevents adenosine degradation, which is an anti-excitatory neuromodulator [11]. Thereby, allopurinol might reduce reperfusion injury and improve outcome in neonates with HIE.

Several preclinical and three small clinical studies in neonates with HIE suggested a possible neuroprotective effect of allopurinol (recently reviewed in Annink et al. [8]). In the first two studies of van Bel et al. and Benders et al. allopurinol was administered within 4 h after birth. Allopurinol improved neurodevelopmental outcome in infants with moderate HIE, but not in severe HIE [12–14]. Gunes et al. administered allopurinol for three days and found improved outcome at one year of age [15]. All three studies were conducted before therapeutic hypothermia became standard of care, so the effect of allopurinol in addition to therapeutic hypothermia has not been investigated yet.

Based on the hypothesis that administration within 4 h after birth was too late to achieve full neuroprotective effect, allopurinol was administered antenatally in case of suspected hypoxia in the antenatal allopurinol trial for reduction of birth asphyxia induced brain damage (ALLO-trial) [16]. In girls, biomarkers as S100B were reduced in the allopurinol group compared to the placebo group. However, there was substantial overtreatment on the one hand and on the other moderately and severely asphyxiated infants were often missed [16].

Consequently, in this study on the effects of *AL*lopurinol in addition to hypothermia treatment for hypoxic-ischemic *B*rain *I*njury on *N*eurocognitive *O*utcome (ALBINO), allopurinol will be administered intravenously within 30 (max. 45) minutes after birth to optimize the timing and inhibition of superoxide formation in asphyxiated infants with evolving HIE.

Importantly, in all antenatal and neonatal studies in HIE, no severe side-effects were seen [12, 13, 15–19]. Also, in other neonatal populations, such as preterm infants and infants with congenital cardiac abnormalities, no severe side effects have been reported following (intravenous or oral) administration of allopurinol [20–24]. In the ALLO-trial, 4.5% of the mothers who received allopurinol had an irritation of the perivascular tissue, caused by the high pH of the allopurinol solution, but this was reversible in all cases [16]. In adults, a rare hypersensitivity reaction to allopurinol has been described after daily administration for a median of two to three weeks [25, 26]. An allopurinol sensitivity reaction in neonates has never been reported and is expected to be extremely unlikely.

## Methods/design

### Trial objectives

The main objective of the ALBINO trial is to evaluate the effect of early postnatal allopurinol administered in addition to standard of care (including therapeutic hypothermia if indicated) on the incidence of death and severe NDI at 24 months of age in newborns with HIE.

Secondly, safety of early postnatal intravenous allopurinol will be evaluated, as well as the pharmacokinetic profile of intravenous allopurinol and the short-term effect of early allopurinol on brain injury assessed by magnetic resonance imaging (MRI) of the brain, cerebral ultrasound, heart function assessed by echocardiography, electro-encephalography (EEG), and biochemical biomarkers.

### Trial design

The ALBINO trial is a European double-blinded randomized placebo-controlled parallel group multicenter trial for superiority of allopurinol versus placebo (mannitol) in addition to therapeutic hypothermia where indicated (Phase III). More than 60 hospitals in ten countries will participate in this study, and ALBINO may expand to additional sites in further countries, after appropriate approvals have been obtained from ethics committees and authorities.

### Population

Term and near-term infants (≥36 weeks) with severe perinatal asphyxia and potentially evolving encephalopathy can be included in the ALBINO trial:

#### Inclusion criteria

Infants must meet at least one of the following five criteria of severe perinatal asphyxia: 1) umbilical or postnatal blood gas within 30 min after birth with a pH < 7.0 or 2) with a base deficit ≥16 mmol/l; 3) need for ongoing cardiac massage at/beyond 5 min postnatally; 4) need for adrenalin administration during resuscitation and/or 5) Apgar score ≤ 5 at 10 min after birth.

Further, the infant must meet two out of the following four criteria for potentially evolving encephalopathy to participate in the study: 1) altered state of consciousness (reduced or absent response to stimulation or hyperexcitability); 2) severe muscular hypotonia or hypertonia; 3) absent or insufficient spontaneous respiration (i.e. gasping only) with need for respiratory support at 10 min postnatally and/or 4) abnormal primitive reflexes (absent suck/gag/ corneal/Moro reflex) or abnormal movements (i.e. potential clinical correlates of seizure activity).

#### Exclusion criteria

Infants will be excluded if the gestational age is below 36 weeks, birth weight is below 2500 g, in the presence of severe congenital malformations or syndromes requiring neonatal surgery or affecting long-term outcome. Furthermore, infants will be excluded if their postnatal age is > 30 min at the end of the screening phase, the neonate is considered “moribund” or “non-viable”, there is a decision of ‘comfort care only’ before study drug administration or if parents decline study participation.

#### Randomization and allocation concealment

Randomization will be performed with randomization software (Randlist Version 1.2) in blocks of four and stratified per center.

Randomization will be performed by allocation of the next consecutive study medication box (including first and second vial of study medication and two vials with sterile water for reconstitution) to an infant.

#### Study intervention

**Fig. 1:**
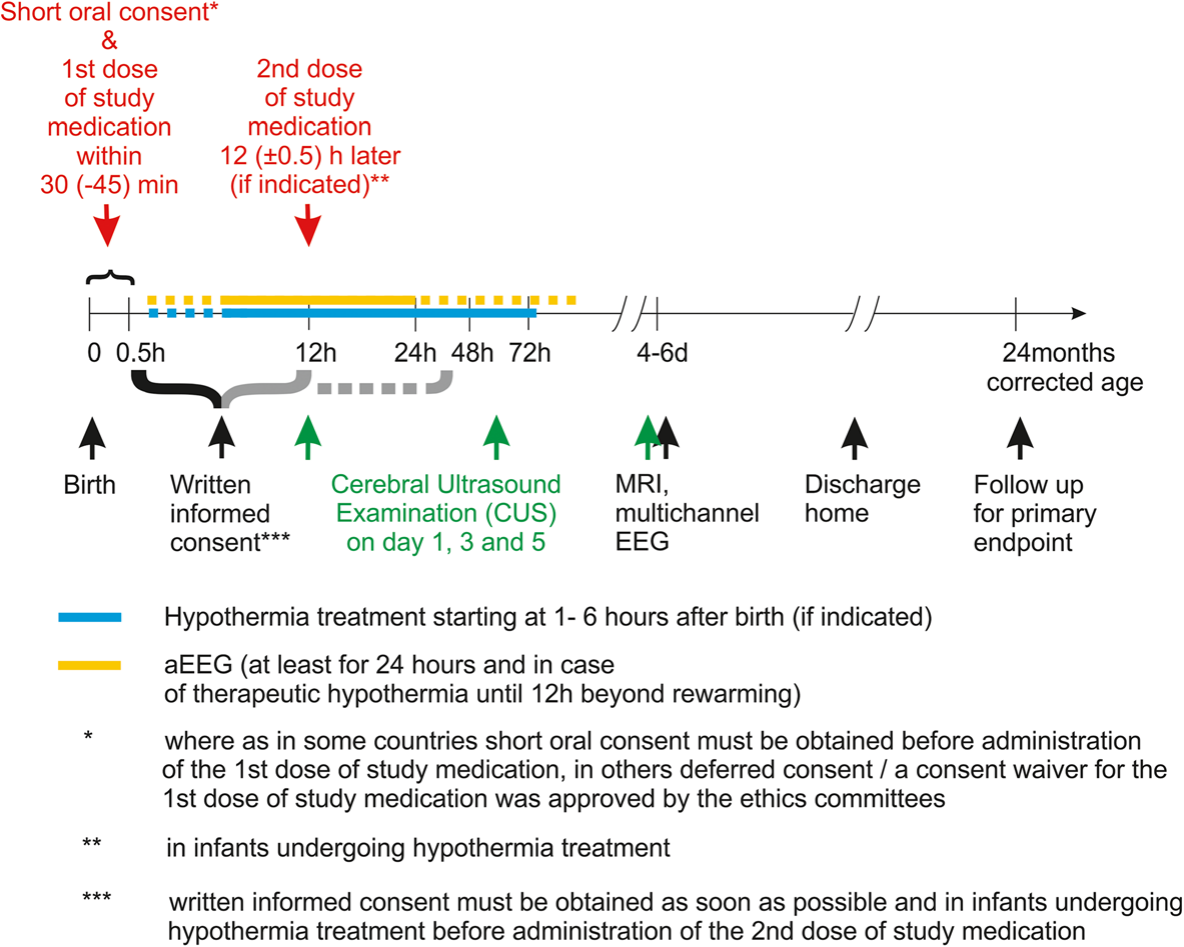
Study interventions in ALBINO

Infants included in the ALBINO trial will receive either allopurinol or placebo (mannitol). Study medication will be administered by intravenous infusion in one or two doses (see Fig. 1). The first dose (20 mg/kg body mass reconstituted in 2.0 ml/kg sterile water for infusion) will be given as soon as possible after birth. The start of infusion of study medication should preferably be within 30 min after birth, but no later than 45 min after birth.

The second dose (10 mg/kg body mass reconstituted in 1.0 ml/kg sterile water for infusion) will be administered 12 h after the first dose. This second dose will only be administered to infants treated with therapeutic hypothermia. Infants who recover quickly and do not qualify for and hence do not undergo hypothermia will not receive a second dose.

Placebo (mannitol) will be given in the same dose, volume and intervals as allopurinol.

#### Concomitant interventions and medication

Any concomitant medication that is medically necessary for the patients will be allowed in the study, except open-label allopurinol in any dosage and any application mode.

Where indicated according to respective national standards or treatment protocols, hypothermia treatment (whole body cooling to 33.5 °C for 72 h) should be started as soon as possible according to local protocols [3, 27].

### Primary outcome

The primary endpoint will be death or severe NDI versus survival without severe NDI at the age of two years. Severe NDI is hereby defined as any of the following: cognitive or language delay defined as a cognitive-composite-score or a language-composite-score on the Bayley Scales of Infant and Toddler Development (3rd edition) < 85 and/or cerebral palsy (CP) according to the Surveillance of Cerebral Palsy in Europe (SCPE) criteria.

### Secondary and further outcomes

The primary endpoint will be reconstituted as dichotomized composite secondary endpoint (survival without NDI versus Death or language-composite-score < 85 or cognitive-composite-score < 85 or CP present). Furthermore, the incidences of death and CP and the composite scores derived from the Bayley test (continuous and dichotomized) as well as the Gross Motor Function Classification Score will be analyzed as secondary outcome variables.

Further important secondary outcome parameters are brain injury assessed by MRI of the brain, cerebral ultrasound, amplitude-integrated EEG, full scale multichannel EEG, heart function assessed by echocardiography, concentrations of peroxidation products and S100B which are markers for brain injury in the blood. Furthermore, pharmacokinetics of allopurinol will be investigated in 48 to 52 patients. Finally, the opinions of parents experiencing two different consent procedures will be evaluated.

### Parental perspectives

Following study participation, parents will be approached again and asked for their opinion on and satisfaction with the consent procedure to inform future investigators in the field of HIE therapy.

### Ethical Considerations

Because allopurinol has to be administered as early as possible after birth to reduce formation of oxygen radicals during reperfusion and because the emergency situation of perinatal asphyxia is very stressful for the parents, the usual procedure of provision of comprehensive oral and written information, time for consideration and full written informed parental consent before study entry is not feasible in the setting of ALBINO. This problem and the various alternative approaches (antenatal consent, short information and oral consent and later full information and written confirmation, waiver of consent for 1st dose and deferred information and consent), have been discussed with external experts on perinatal HIE as well as medical ethicists and a balance between the need to inform the parents and the feasibility of the study was sought in collaboration with the relevant ethics committees in each participating country.

### Community Engagement

Information material, such as posters and flyers, that provides short information for parents, will be available in prenatal clinics and delivery areas and will direct interested parents to the study home page (www.albino-study.eu).The homepage will grant access to nationally approved full parent information material. A press release will inform the community around study sites about the ALBINO study.

Parents, who do not want to participate in the ALBINO-trial, will have the option to deny participation even before delivery verbally or on a ‘declaration of intent’-form printed on the flyers informing about the study. This can be completed and kept in the maternal health passport to inform the staff in the delivery room.

### Form of Consent

According to the relevant ethics committee’s decisions, either a deferred consent or an initial short oral consent approach will be used for obtaining parental consent.

The deferred consent procedure has previously been used in emergency research in adults and is in compliance with §30 of the Declaration of Helsinki (Fortaleza 2013 [28]). In the case that a child fulfills the inclusion criteria and meets no exclusion criterion, physicians will administer the first dose of study medication in the delivery room without prior consent (i.e., a ‘consent waiver’ was granted for the 1st dose of study medication). Parents will receive detailed information later and will be asked for written informed consent for continued participation in the study (as soon as possible, at the latest before the 2nd dose of study medication if indicated). The deferred consent procedure has been approved in Austria, Belgium, Estonia, Finland and Norway.

In Germany, the Netherlands, Italy, Switzerland and Spain, the ethical committees did not agree on the deferred consent procedure, so in these countries the short oral consent procedure will apply: short oral information (duration < 5 min) on the indication and the potential benefits and risks of the study medication must be provided to at least one parent and oral (or written) consent of this parent must be obtained before the 1st dose of study medication can be administered. Again, both parents will receive more detailed information and will be asked for full written consent as soon as possible and at the latest before the 2nd dose of study medication will be administered (if indicated).

### Statistical analysis

#### Sample size, power and study duration

The primary assessment for efficacy will compare the proportions of infants surviving without severe NDI versus those of infants who died or survived with severe NDI at the age of two years.

**Fig. 2:**
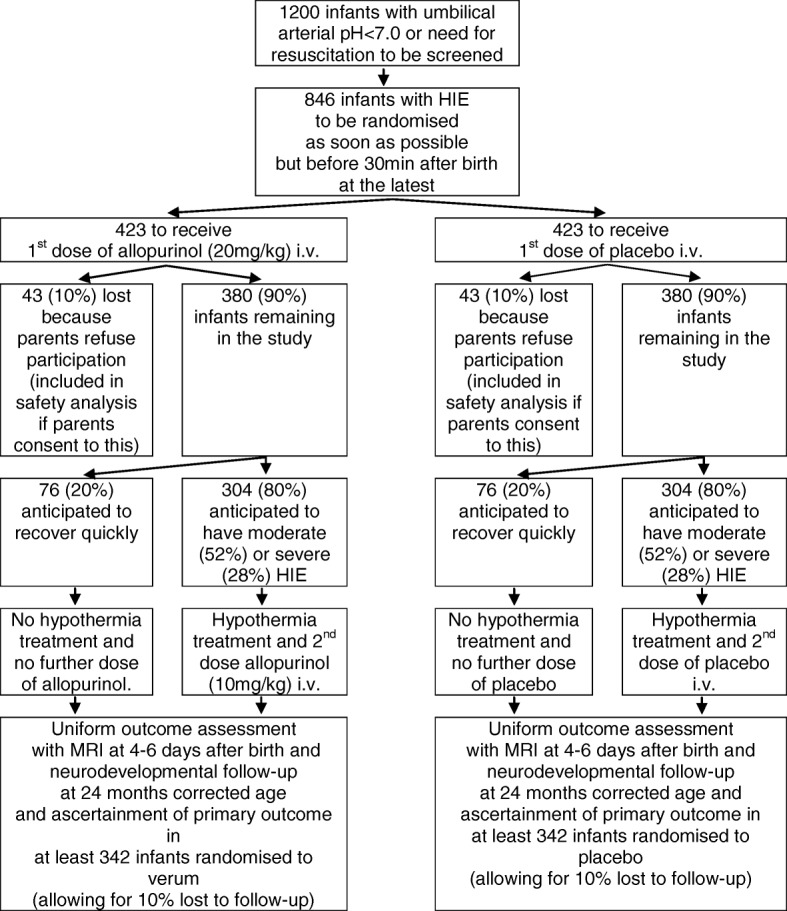
Anticipated Trial Flow

Based on the above referenced (preliminary) clinical studies from the pre-therapeutic hypothermia era and clinical studies on hypothermic treatment, it is estimated that the combined incidence of death and severe NDI in the control group will be 37 and 27% in the allopurinol group. Therefore, we calculated with a two-sided X^2^-test (alpha = 0.05, power 80%) a sample size of 682 infants (341 per treatment group) in which the primary outcome should be ascertained. Assuming a drop-out rate of 10% for loss to follow-up, a total of 760 infants need to be enrolled. And assuming that 10% of the parents will refuse participation after the initial dose of the study drug, 846 infants have to be randomized (see Fig. 2).

We estimate a recruitment of about 35 patients per month in approximately 70 study centers (the recruitment of additional study sites is ongoing) and therefore recruitment will last 24 months.

### Data analysis

All statistical analyses will be described in detail in a statistical analysis plan completed before closure of the database.

### Monitoring safety

An independent Data Monitoring Committee (DMC) will monitor the participants’ well-being and the overall risk/benefit-ratio of the study. National monitors will monitor the accuracy and completeness of the data and the safety issues such as the presence of serious adverse events.

### Regulatory aspects

#### Trial sponsor

Sponsor of the ALBINO-trial is the University Hospital Tuebingen, Geissweg 3, 72076 Tuebingen, Germany. Contact is available under albino@med.uni-tuebingen.de.

#### Orphan Drug Designation

The Committee for Orphan Medicinal Products (COMP) has given a positive advice to ACE Pharmaceuticals for the orphan drug designation for allopurinol sodium for treatment of perinatal asphyxia (EU/3/15/1493) and an Orphan Drug Designation has been granted by the European Medicines Agency. The public summary is available at: https://www.ema.europa.eu/documents/ orphan-designation/eu/3/15/1493-public-summary-opinion-orphan-designation-allopurinol-sodium-treatment-perinatal-asphyxia_en.pdf.

#### Scientific Advice from the European Medicines Agency

In November 2015, ACE Pharmaceuticals has requested Scientific Advice and Protocol Assistance from the European Medicines Agency, including questions specifically related to the study protocol and the intended procedure of deferred consent. Written scientific advice was received in May 2016 and after careful consideration by the Steering Committee, the relevant issues were subsequently incorporated into the study protocol.

#### Medical ethics committees

At the time of publication, the relevant ethics committees in ten European countries approved the study with either the short oral consent procedure or the deferred consent procedure. Applications for approvals are currently underway in two additional countries.

#### National Regulatory/Competent Authorities

At the time of publication, eleven European National Regulatory/Competent Authorities approved the study. Application for approval is currently underway in one additional country.

## Discussion

ALBINO is a randomized controlled trial investigating the safety and efficacy of allopurinol in (near-) term infants with HIE.

A decision was made for a large phase III trial for efficacy and safety because preliminary clinical data from postnatal and prenatal allopurinol trials already suggested a reduction in brain injury by allopurinol without apparent adverse effects. Another small proof-of-principle or dose seeking study would have added little with respect to safety and clinically relevant outcomes. Survival without NDI was selected as the primary endpoint of this study, because this outcome parameter is most meaningful to the children and their families.

The calculated starting dose was based on previous studies: the doses used in the first studies with allopurinol in neonates undergoing extracorporeal membrane oxygenation and in neonates diagnosed with hypoplastic left heart syndrome (10 and 30 mg/kg birth weight respectively) gave 100% xanthine-oxidase inhibition [21, 23]. Higher concentrations may be needed for the iron chelating and reactive oxygen scavenging effect of allopurinol. Even with higher doses (up to 40 mg/kg birth weight per day for 3 days) no adverse effects were seen, with special attention to skin rashes and leukopenia [15].

A significant beneficial effect of allopurinol in moderately asphyxiated neonates has been found on long-term (4–5 years) neurodevelopmental outcome by Kaandorp et al. (2012), which was a meta-analyses of the study from van Bel et al. (1998) and Benders et al. (2006) [12, 13]. These latter trials administered 2 times 20 mg/kg birth weight allopurinol with 12 h interval. The doses of the ALBINO trial are based on these three studies. However, pharmacokinetics of allopurinol during hypothermia have not yet been determined in neonates with HIE. The second dose in the ALBINO study (only during hypothermia) is adjusted for the hypothermia treatment which may possibly slow-down allopurinol and oxypurinol metabolism and elimination. In the latter case this would lead to higher circulating concentrations of allopurinol and, respectively, oxypurinol.

In previous studies the plasma concentrations of allopurinol were often supra-therapeutic without any side effect [19, 29]. These supra-therapeutic levels seem to be important for the direct scavenging of hydroxyl and free iron by allopurinol. However, to ensure that in addition to therapeutic hypothermia plasma concentrations are not lower than in the earlier clinical trials indicating efficacy, blood sampling for pharmacokinetic analyses will be performed in 48 to 52 infants (in selected centers) recruited during the first year of the study and may lead to adaptation of doses.

Mannitol is used as placebo, since its freeze-dried white powder and the reconstituted solution, have the same visual aspects and volume as the freeze-dried sodium salt of allopurinol and its reconstitution solution (10 ml of a colorless, clear solution in a 20 ml vial). The dosage of mannitol is 50 times lower than the dose of mannitol used for neuroprotection [30], and a normal daily dose of intravenous paracetamol will include more mannitol as supporting agent than the dose administered in ALBINO (i.e. 100 ml solution for injection contains 1000 mg acetaminophen and 3670-3850 mg mannitol [31, 32]. For each single dose of 12.5 mg/kg paracetamol i.v. [33], 45.9–48.1 mg/kg of Mannitol are concomitantly administered).

Inclusion and exclusion criteria were selected to recruit a patient population similar to the TOBY trial of whole body cooling [3], but took into account that the assessment for eligibility has to be done much earlier, i.e., within 30 min after birth.

The ALBINO study group extensively discussed the various ethical implications of need for additional treatment for HIE, need to administer allopurinol very early for best efficacy, need for parental consent to ensure patient autonomy and burden to the parents in the emergency situation of perinatal HIE.

The European Foundation for the Care of Newborn Infants (EFCNI), which is composed of parents, healthcare experts, scientists and politicians, has been asked for advice. The EFCNI endorsed the conduct of the ALBINO trial in a letter of support in September 2016. Because perinatal HIE occurs rarely and unpredictably and because of the need for very early administration of allopurinol, the EFCNI agreed with the approach of deferred consent.

Furthermore, independent ethics experts provided advice. Whereas all experts agreed that regular informed consent by the parents, which includes appropriate time for reflection and further questions is not feasible before administration of the 1st dose of study medication in the context of ALBINO due to the unpredictable emergency situation. Opinions within the group as well as among external experts ranged from ‘deferred consent is unacceptable’ to ‘deferred consent is justified and the better option’, so that the decision was left to the national leading ethics committees in each country.

Currently, we are conducting a survey among parents-to-be and parents of infants with a history of HIE to better understand how parents might feel about deferred versus short oral consent. An additional survey will follow parents of infants enrolled in the ALBINO study to capture their satisfaction with the various approaches and to inform future trials in similar situations.

In conclusion, infants with HIE still suffer from death and long-term NDI despite improved standards of care including therapeutic hypothermia. The neurodevelopmental outcome of infants with HIE should be further improved with additional neuroprotective interventions. The aim of the ALBINO trial is to investigate the neuroprotective effect of very early allopurinol within 45 min after birth aiming to reduce the formation of the toxic superoxide and subsequent secondary energy failure and apoptosis.

## Trial status

Protocol version 5: 19. December 2017. Recruitment has started in April 2018 and is expected to be finalized in April 2020. The last patient out (after follow-up) will then be expected in April 2022.

## Data Availability

Data sharing is not applicable to this article as no datasets were generated or analyzed during the current study.

## Abbreviations

ALLO-trial: Antenatal allopurinol trial for reduction of birth asphyxia induced brain damage
CP: Cerebral Palsy
COMP: Committee for Orphan Medicinal Products
DMC: Data Monitoring Committee
ALBINO: Effect of Allopurinol in addition to hypothermia for hypoxic-ischemic brain injury on neurocognitive outcome
EEG: Electro-encephalography
EFCNI: European Foundation for the Care of Newborn Infants
HIE: Hypoxic-ischemic Encephalopathy
MRI: Magnetic Resonance Imaging
NDI: Neurodevelopmental Impairment
SCPE: Surveillance of Cerebral Palsy in Europe
TOBY: Total Body Hypothermia for Neonatal Encephalopathy Trial
